# The association between the duration of fluoropyrimidine-based adjuvant chemotherapy and survival in stage II or III gastric cancer

**DOI:** 10.1186/s12957-016-0845-1

**Published:** 2016-04-02

**Authors:** Seong-Geun Kim, Sun-Hwi Hwang

**Affiliations:** Department of Internal Medicine, Pusan National University Yangsan Hospital, Beomeo-ri Mulgeum-eup, Yangsan, Gyeongnam 626-770 South Korea; Department of Surgery, Pusan National University Yangsan Hospital, Beomeo-ri Mulgeum-eup, Yangsan, Gyeongnam 626-770 South Korea; Research Institute for Convergence of Biomedical Science and Technology, Pusan National University Yangsan, Yangsan, South Korea; Division of Gastric Surgery, Department of Surgery, Pusan National University Yangsan Hospital, Beomeo-ri Mulgeum-eup, Yangsan, Gyeongnam 626-770 South Korea

**Keywords:** Fluoropyrimidine based, Survival, Gastric cancer

## Abstract

**Background:**

This study was conducted to propose the optimal duration of fluoropyrimidine-based adjuvant chemotherapy consisting of fluoropyrimidine derivatives alone or combined with intravenous platinum for stage II or III gastric cancer (GC).

**Methods:**

We analyzed retrospectively the data from 2219 patients with histologically confirmed adenocarcinoma in the stomach, who underwent a curative gastrectomy with lymphadenectomy from 2005 to 2012. Five-year overall survival (OS) and 3-year relapse-free survival (RFS) were analyzed according to the duration of fluoropyrimidine-based adjuvant chemotherapy.

**Results:**

Data from 617 patients with stage II or III GC were analyzable; 187 patients (30.3 %) were treated with surgery alone, while 430 patients (69.7 %) were treated with postoperative adjuvant chemotherapy. The duration of adjuvant chemotherapy was less than 6 months [group 1] in 147 patients (34.2 %), 6 months to less than 12 months [group 2] in 94 patients (21.9 %), 1 year to less than 2 years [group 3] in 139 patients (32.3 %), and over 2 years [group 4] in 50 patients (11.6 %). The 5-year OS in groups 1, 2, 3, and 4 was 75.7, 87, 90.3, and 93.4 %, respectively, while 3-year RFS was 52.5, 58.8, 81.4, and 94.0 %, respectively.

**Conclusions:**

In this retrospective study, we did not demonstrate any significant improvement in OS and RFS by longer periods of fluoropyrimidine-based adjuvant chemotherapy in stage II or III GCs. Further prospective randomized studies are needed.

## Background

According to the estimated incidence, mortality and worldwide prevalence data for 2012 from the International Agency for Research on Cancer, gastric cancer (GC) is the fifth most common malignancy in the world. With nearly 1 million cases per annum, it is the third leading cause of cancer-related death in both sexes worldwide [1]. In addition, GC is the second most common malignancy in Asia and more than half of new GCs occur in Eastern Asia [2]. At present, adjuvant chemotherapy or chemoradiotherapy after surgical resection of GC is a reasonable option because high rates of locoregional or distant recurrences have been reported [3–6].

For the treatment of gastric and gastroesophageal junction adenocarcinoma, the Intergroup trial 0116 (INT-0116) in 2001 showed the first high-level evidence for improved survival from adjuvant therapy in GC [7]. Nowadays, extensive (D2) lymph-node dissection is recommended because the adequacy of surgical resection is an important issue. However, only 10 % of patients underwent D2 dissection, while 36 % had D1 dissection, and 54 % had D0 lymphadenectomy in this trial. The median overall survival (OS) in the surgery-only group was 27 months, as compared to 36 months in the adjuvant chemoradiotherapy group. Since then, postoperative chemoradiotherapy has become the standard treatment after a curative resection in the USA. In 2006, the Medical Research Council Adjuvant Gastric Infusional Chemotherapy (MAGIC) trial randomly assigned patients with resectable stomach, esophagogastric junction, or lower esophagus cancer to either perioperative chemotherapy following surgery (250 patients) or surgery alone (253 patients). The primary endpoint was OS. Compared to the surgery group (23 %), the perioperative chemotherapy group (36 %) had a higher likelihood of 5-year overall survival. As the MAGIC trial shows a survival benefit, in Europe, perioperative chemotherapy with epirubicin, cisplatin, and 5-fluorouracil (ECF) has become the standard of care following curative resection [4].

S-1 has been developed mainly in Japan because the pharmaceutical company producing S-1 is a domestic Japanese company, and there are sufficient numbers of patients with gastric cancer in Japan for clinical trials. Phase II trials of S-1 monotherapy (40 mg/m^2^, twice a day, on days 1–28, every 6 weeks) were conducted in Japan [8]. In 1999, the Japanese government approved this drug for treating gastric cancer on the basis of the results of the two domestic phase II trials [9]. S-1 is a novel oral agent containing tegafur, a prodrug of 5-fluorouracil (5-FU), and two biochemical modulators of 5-FU including 5-chloro-2,4-dihydroxypyridine and potassium oxonate. 5-Chloro-2,4-dihydroxypyridine increases the pharmacological action of 5-FU by inhibiting dihydropyrimidine dehydrogenase. Potassium oxonate, which localizes to the mucosal cells of the gastrointestinal (GI) tract after oral administration, reduces GI toxicity by suppressing the activation of 5-FU in the GI tract. In Japan, the patients with stage II or III GC who underwent gastrectomy plus extended (D2) lymph node dissection were randomly assigned to either the surgery with S-1 adjuvant therapy group or to the surgery alone group. The primary endpoint was OS. The 3-year OS rate was 80.1 % in the S-1 group and 70.1 % in the surgery-only group. The hazard ratio (HR) for death in the S-1 group compared to the surgery-only group was 0.68. The OS rate at 5 years was 71.7 % in the S-1 group and 61.1 % in the surgery-only group. The rate of relapse-free survival (RFS) at 3 years was 72.2 % in the S-1 group and 59.6 % in the surgery-only group. The 5-year RFS rate was 65.4 % in the S-1 group and 53.1 % in the surgery-only group [5]. The CLASSIC study was an open-label, phase 3, randomized controlled trial undertaken in 37 centers in South Korea, China, and Taiwan. Patients with stage II, IIIA, and IIIB GC who had undergone curative D2 gastrectomy were randomly assigned to either adjuvant chemotherapy with capecitabine plus oxaliplatin (XELOX) for 6 months or surgery only. The primary endpoint was 3-year disease-free survival (DFS). The 3-year DFS was 74 % in the chemotherapy after surgery group and 59 % in the surgery-only group. The 5-year OS rate was 78 % in the XELOX group and 69 % in the surgery alone group. The 5-year DFS rate was 68 % in the XELOX group and 53 % in the surgery alone group. In this study, more than half of the patients who received chemotherapy had peripheral neuropathy, which is a cumulative, dose-related toxic effect associated with oxaliplatin, but grade 3 or 4 events were infrequent [6].

Among the regimens mentioned above, most randomized prospective studies have evaluated the effects of chemotherapy over periods ranging from 6 to 12 months. Postoperative FU-based oral chemotherapy, such as S-1 for 1 year or capecitabine plus oxaliplatin for 6 months, is proven as the effective treatments for localized GC after D2 gastrectomy [5, 6]. However, it is difficult to say which regimens are better for adjuvant chemotherapy of GC because they have similar efficacies and different toxicities. Although only appropriately designed and powered randomized clinical trials can address the optimal duration of adjuvant chemotherapy, because of the relatively high recurrence rate in GC patients, ethical concerns are likely to prevent any prospective study of the optimal duration of adjuvant treatment from being undertaken [10].

Consequently, we wanted to undertake a retrospective analysis exploring the correlation between adjuvant chemotherapy duration and OS or RFS in advance of conducting a randomized prospective study.

## Methods

### Patients

The data were collected from 2219 patients with histologically confirmed adenocarcinoma in the stomach, who underwent curative gastrectomy with lymphadenectomy from 2005 to 2012 in Pusan National University Hospital. The baseline patient characteristics are shown in Table 1.

**Table 1:** Patient and tumor characteristics

	Surgery only	Adjuvant CT	*p* value
	*N* = 187	*N* = 430	
Sex			0.911
M	127 (67.9)	294 (68.4)	
F	60 (32.1)	136 (31.6)	
Age (median, years)			0.015
	70 (31~96)	66 (29~90)	
<65	58 (31.0)	193 (45.0)	0.001
≥65	129 (69.0)	236 (55.0)	
Performance status (ECOG)			0.387
0	181 (96.8)	422 (98.1)	
1	4 (2.1)	6 (1.4)	
2	1 (0.5)	2 (0.5)	
3	1 (0.5)	0	
Preoperative CEA (ng/mL)	2.2 (0.2~98.8)	2.4 (0.2~169.1)	0.710
Scope of LN dissection			0.004
D1	23 (12.5)	31 (7.2)	
D1 + A	1 (0.5)	23 (5.4)	
D1 + B	5 (2.7)	23 (5.4)	
D2	151 (82.1)	347 (81.1)	
D3	4 (2.2)	4 (0.9)	
Histology			0.209
Differentiated	88 (47.1)	226 (52.6)	
Undifferentiated	99 (52.9)	204 (47.4)	
Lymphatic invasion			0.948
No	60 (32.1)	136 (31.6)	
Yes	127 (67.9)	293 (68.1)	
Unknown	0	1 (0.2)	
Depth of tumor invasion (AJCC 7th)			
T stage			0.073
T1a	3 (1.6)	3 (0.7)	
T2	26 (13.9)	69 (16.05)	
T3	91 (48.7)	165 (38.4)	
T4a	58 (31.0)	160 (37.2)	
T4b	8 (4.3)	24 (5.6)	
N stage			<0.001
N0	55 (29.4)	70 (16.28)	
N1	48 (25.7)	104 (24.19)	
N2	48 (25.7)	108 (25.12)	
N3a	25 (13.4)	88 (20.47)	
N3b	11 (5.9)	60 (13.95)	
Stage			0.002
IIA	74 (39.6)	102 (23.72)	
IIB	35 (18.7)	89 (20.7)	
IIIA	27 (14.4)	67 (15.58)	
IIIB	24 (12.8)	92 (21.39)	
IIIC	27 (14.4)	78 (18.60)	

*CT* chemotherapy

### Study design and treatment

**Fig. 1:**
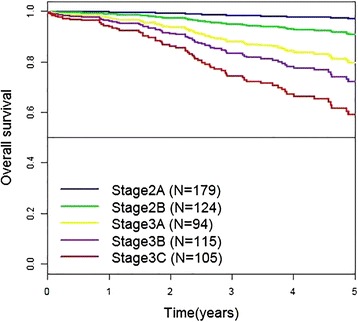
Kaplan–Meier curves for overall survival according to cancer stages in patients treated with adjuvant chemotherapy

This study is a retrospective study for evaluating the optimal duration of fluoropyrimidine-based adjuvant chemotherapy in patients who had initially operable stage II or III GC. The adjuvant chemotherapy consisted of fluoropyrimidine derivatives (doxifluridine, UFT, S-1, capecitabine) alone or combined with platinum (cisplatin or oxaliplatin). Patients with stage II or III GC were divided into five stages according to the *American Joint Committee on Cancer staging manual*, *7th edition* [Fig. 1] [11].

### Statistical methods

The primary analysis involved evaluating the association between the duration of chemotherapy and OS, which was defined from the time of surgery to death or the last follow-up visit. Secondary analysis included 3-year RFS, which was calculated as the time from surgery to the time of recurrence. Univariate and multivariate analyses using a Cox proportional hazard regression model were carried out, and hazard ratios (HR) were estimated with 95 % confidence interval (95 % CI) limits. Both univariate and multivariate analyses were conducted to establish the association between prognosis and age, sex, depth of tumor invasion, lymph node metastasis, histological type, stage, and the duration of adjuvant chemotherapy. Survival curves were generated by the Kaplan–Meier method. A *p* value of less than 0.05 was considered significant. All analyses were performed using SPSS for Windows, version 21.0 (SPSS Inc., Chicago, IL, USA), and R software, version 3.1.1 (R Foundation for Statistical Computing).

## Results

After patients with any unanalyzable conditions were excluded, 617 patients with stage II or III GC were enrolled. Due to patients’ refusal or postoperative morbidity, 187 patients (30.3 %) were treated with surgery alone; 430 patients (69.7 %) were treated for diverse durations with various fluoropyrimidine-based adjuvant chemotherapy regimens. The numbers of male patients were 294 (68.4 %) and the numbers of female patients were 136 (31.6 %), and the numbers of patients below the age of 60, between the ages of 60 and 70, and over the age of 70 who underwent adjuvant chemotherapy were 118 (27.5 %), 125 (29.1 %), and 186 (43.4 %), respectively. The median follow-up duration of OS was 42.2 months (mean = 41.9, SD = 26.2); the median follow-up duration of RFS was 14.5 months (mean = 19.4, SD = 16.7).

**Fig. 2:**
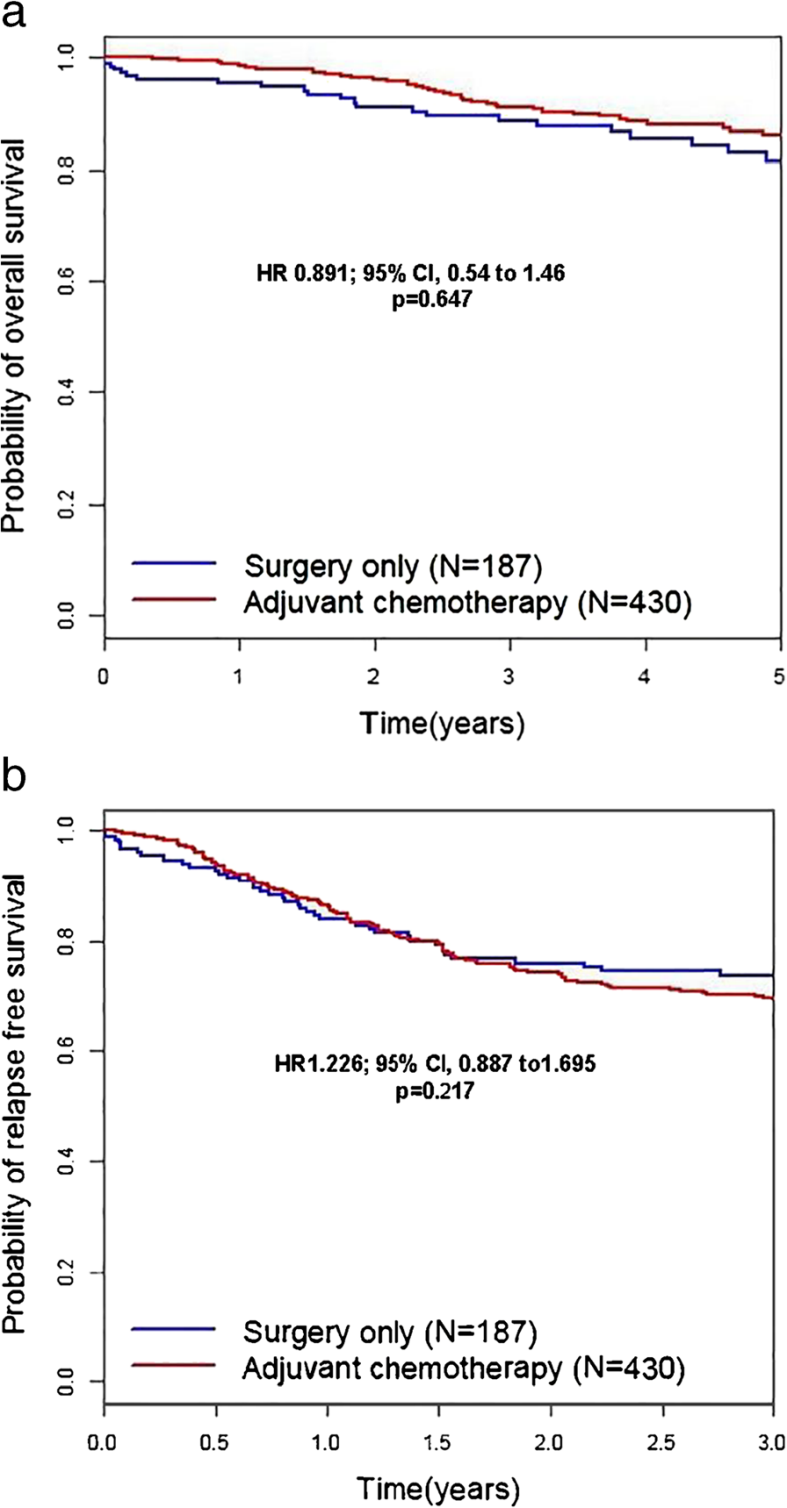
Kaplan–Meier curves for overall survival (**a**) and relapse-free survival (**b**). The rates of overall survival in the adjuvant chemotherapy and surgery-only groups are 86.0 and 81.4 %, respectively, while the rates of relapse-free survival are 69.3 and 73.9 %, respectively. The hazard ratio for death in the adjuvant chemotherapy group as compared to the surgery-only group is 0.891 (95 % confidence interval 0.54–1.46, *p* = 0.647). The hazard ratio for relapse in the adjuvant chemotherapy group is 1.226 (95 % confidence interval 0.887–1.695, *p* = 0.217). *p* values were calculated using the stratified log-rank test

The 5-year OS rates for the adjuvant chemotherapy and surgery-only groups were 86.0 and 81.4 %, respectively. The hazard ratio (HR) for death in the adjuvant chemotherapy group as compared to the surgery-only group was 0.891, with a 95 % confidence interval (CI) of 0.54–1.46 (*p* = 0.647). However, the 3-year RFS rate for the adjuvant chemotherapy group was 69.3 % compared to 73.9 % in the surgery-only group. The HR for relapse in the adjuvant chemotherapy group was 1.226 (95 % CI 0.887–1.695, *p* = 0.217) [Fig. 2].

The duration of adjuvant chemotherapy was less than 6 months [group 1] in 147 patients (34.2 %), 6 months to less than 12 months [group 2] in 94 patients (21.9 %), 1 year to less than 2 years [group 3] in 139 patients (32.3 %), and over 2 years [group 4] in 50 patients (11.6 %) in Table 2.

**Table 2:** The proportions according to the lengths of chemotherapy

Durations of chemotherapy	*n* (%)
<6 M	147 (34.2 %)
6~12 M	94 (21.9 %)
12~24 M	139 (32.3 %)
>24 M	50 (11.6 %)

*n* number of patients, *M* months

Adjuvant chemotherapy regimens for GC in this study were divided into seven categories depending on the administration method, which included intravenous (IV), per oral (PO), or a combination of IV and PO methods. The most commonly used drugs were doxifluridine, UFT, and S-1 in order of the decreasing frequency. The median cycles of the regimens are also listed in Table 3. The distribution of patients according to durations of chemotherapy and TNM stages is shown in Table 4.

**Table 3:** The names and median cycles of regimens used as adjuvant chemotherapy

Regimens	Number	Percent	Cycles
Doxifluridine (D1–28) q 4w	205	47.7	19.9
UFT (D1–28) q 4w	57	13.3	11.9
S-1 (D1–28) q 6w	51	11.9	5.0
Capecitabine (D1–14) + cisplatin (D1) q 3w	48	11.2	6.8
5-FU (D1–5) + cisplatin (D1) q 3w	44	10.2	5.7
S-1 (D1–14) + cisplatin (D1)	18	4.2	5.7
Oxaliplatin (D1) + leucovorin (D1–2) + 5-FU (D1–2) q 2w	7	1.6	12.6

*n* number of patients, *q* every, *w* weeks, *D* day

**Table 4:** The distribution of patients according to durations of chemotherapy and TNM stages

Stage (TNM)	Durations of chemotherapy			
	<6 M	6~12 M	12~24 M	>24 M
IIA	25 (17.0 %)	13 (13.8 %)	46 (33.0 %)	18 (36.0 %)
IIB	21 (14.3 %)	21 (22.3 %)	34 (24.4 %)	13 (26.0 %)
IIIA	24 (16.3 %)	14 (14.9 %)	22 (15.8 %)	7 (14.0 %)
IIIB	39 (26.5 %)	21 (22.3 %)	23 (16.5 %)	9 (18.0 %)
IIIC	38 (25.9 %)	25 (26.6 %)	14 (10.0 %)	3 (6.0 %)

Numbers in parenthesis represent the row percentages

*M* months

**Fig. 3:**
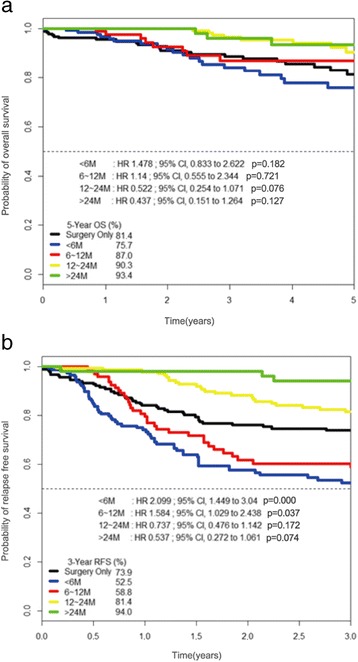
Kaplan–Meier curves for overall survival (**a**) and relapse-free survival (**b**) according to adjuvant chemotherapy durations in patients with gastric cancer. The duration of adjuvant chemotherapy was less than 6 months [group 1] in 147 patients (34.2 %), from 6 months to less than 12 months [group 2] in 94 patients (21.9 %), from 1 year to less than 2 years in [group 3] 139 patients (32.3 %), and over 2 years [group 4] in 50 patients (11.6 %). The reference category of Cox regression analysis in Fig. 3a, b is no adjuvant treatment or surgery-only group. Five-year OS rates for groups 1, 2, 3, and 4 were 75.7 % (HR 1.478, 95 % CI 0.833–2.62, *p* = 0.182), 87 % (HR 1.140, 95 % CI, 0.555–2.344, *p* = 0.721), 90.3 % (HR 0.522, 95 % CI 0.254–1.071, *p* = 0.076), and 93.4 % (HR 0.437, 95 % CI 0.151–1.264, *p* = 0.127), respectively. The 3-year RFS rates for groups 1, 2, 3, and 4 were 52.5 % (HR 2.099, 95 % CI 1.449–3.040, *p* = 0.000), 58.8 % (HR 1.584, 95 % CI 1.029–2.438, *p* = 0.037), 81.4 % (HR 0.737, 95 % CI 0.476–1.142, *p* = 0.172), and 94.0 % (HR 0.537, 95 % CI 0.272–1.061, *p* = 0.074), respectively [Fig. 3]. *p* values were calculated with the use of the stratified log-rank test

**Fig. 4:**
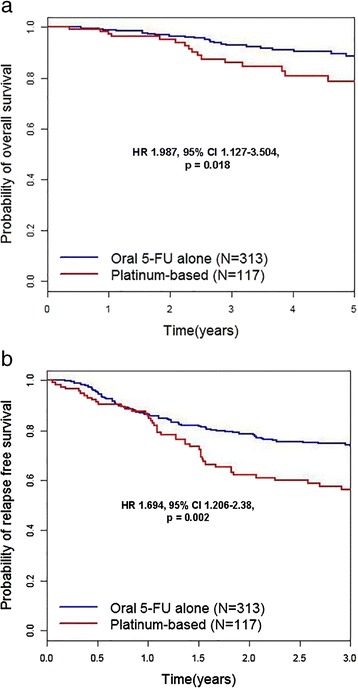
Kaplan–Meier curves for overall survival (**a**) and relapse-free survival (**b**) in the oral 5-fluorouracil alone group (*n* = 313) versus the platinum-based group (*n* = 117). The oral 5-fluorouracil-alone group has better overall survival compared to the platinum-based group (hazard ratio 1.987, 95 % confidence interval 1.127–3.504, *p* = 0.018) and relapse-free survival (hazard ratio 1.694, 95 % confidence interval 1.206–2.38, *p* = 0.002). *p* values were calculated using the stratified log-rank test

For subgroup analysis, the 5-year OS and 3-year RFS rates were analyzed according to the duration of adjuvant chemotherapy. The reference category of Cox regression analysis in Fig. 3a, b is no adjuvant treatment or surgery-only group. Five-year OS rates for groups 1, 2, 3, and 4 were 75.7 % (HR 1.478, 95 % CI 0.833–2.62, *p* = 0.182), 87 % (HR 1.140, 95 % CI, 0.555–2.344, *p* = 0.721), 90.3 % (HR 0.522, 95 % CI 0.254–1.071, *p* = 0.076), and 93.4 % (HR 0.437, 95 % CI 0.151–1.264, *p* = 0.127), respectively. The 3-year RFS rates for groups 1, 2, 3, and 4 were 52.5 % (HR 2.099, 95 % CI 1.449–3.040, *p* = 0.000), 58.8 % (HR 1.584, 95 % CI 1.029–2.438, *p* = 0.037), 81.4 % (HR 0.737, 95 % CI 0.476–1.142, *p* = 0.172), and 94.0 % (HR 0.537, 95 % CI 0.272–1.061, *p* = 0.074), respectively [Fig. 3]. Compared to long-term administration of oral 5-FU chemotherapy alone, regimens combined with platinum had a hazardous effect on OS (HR 1.987, 95 % CI 1.127–3.504, *p* = 0.018) and RFS (HR 1.694, 95 % CI 1.206–2.38, *p* = 0.002) [Fig. 4]. *p* values were calculated using the stratified log-rank test.

Multivariate analyses of OS revealed the prognostic significance of the depth of tumor invasion, lymph node metastasis, and 12- to 24-month duration of adjuvant chemotherapy in Table 5. Multivariate analyses of RFS also showed the prognostic significance of the depth of tumor invasion, lymph node metastasis, histological type, and periods of adjuvant chemotherapy over 12 and 24 months in Table 6.

**Table 5:** Univariate and multivariate analyses of prognostic factors for overall survival according to the period of adjuvant chemotherapy

Factors (OS)	Univariate analysis	Multivariate analysis		
	*p* value	*p* value	Hazard ratio	95 % CI
Age				
<65 vs. >65 years	.154	.094	0.663	0.410–1.073
Sex				
Male vs. female	.596	.302	0.757	0.447–1.284
Depth of tumor invasion				
t1, 2 vs. t3, 4	<.001	.014	5.176	1.390–19.269
Lymph node metastasis				
n0, 1 vs. n2, 3	<.001	.019	4.819	1.288–18.022
Histological type (WHO)				
Undifferentiated vs. differentiated	.348	.703	0.547	0.362–1.985
Lauren classification				
Diffuse vs. intestinal	.170	.556	1.271	0.572–2.825
Stage				
II vs. III	<.001	.851	1.142	0.286–4.559
Period of adjuvant chemotherapy				
<6 months	.182	.374	0.877	0.476–1.616
6~12 months	.721	.267	0.655	0.310–1.383
12~24 months	.076	.005	0.347	0.167–0.726
>24 months	.127	.061	0.358	0.123–1.047

*CI* confidence interval, *OS* overall survival

**Table 6:** Univariate and multivariate analyses of prognostic factors for relapse-free survival according to the period of adjuvant chemotherapy

Factors (RFS)	Univariate analysis	Multivariate analysis		
	*p* value	*p* value	Hazard ratio	95 % CI
Age				
<65 vs. >65 years	.667	.485	1.110	0.829–1.485
Sex				
Male vs. female	.831	.820	0.965	0.708–1.315
Depth of tumor invasion				
t1, 2 vs. t3, 4	<.001	.005	2.382	1.304–4.353
Lymph node metastasis				
n0, 1 vs. n2, 3	<.001	.016	2.128	1.154–3.927
Histological type (WHO)				
Undifferentiated vs. differentiated	.904	.015	0.516	0.303–0.881
Lauren classification				
Diffuse vs. intestinal	.050	.012	1.936	1.158–3.237
Stage				
II vs. III	<.001	.115	1.706	0.877–3.319
Period of adjuvant chemotherapy				
< 6 months	<.001	.049	1.481	1.001–2.192
6~12 months	.037	.487	1.171	0.750–1.829
12~24 months	.172	.022	0.593	0.379–0.926
>24 months	.074	.018	0.438	0.221–0.870

*CI* confidence interval, *RFS* relapse-free survival

## Discussion

In order to improve OS or RFS with adjuvant chemotherapy, both duration and cumulative dose intensity of adjuvant treatment are as important as the regimen. As the optimal length of time for adjuvant chemotherapy has not been established, if adjuvant chemotherapy does not adversely affect patients, continuation of chemotherapy over the planned period should be considered. Therefore, in Japan, a retrospective study was conducted to evaluate the effectiveness of oral anti-cancer drugs (for 2 years) as postoperative adjuvant chemotherapy in GC patients [12]. Authors divided the 20 years chronologically into the UFT (5-FU analog, tegafur combined with uracil in a ratio of 1:4, Taiho Pharmaceutical Co. Ltd., Tokyo, Japan) term (1989–2003) and the S-1 term (2004–2008). The patients from each term were then divided into three groups according to the length of drug administration, namely, the surgery-alone group, the 1-year group, and the 2-year group. The survival time of the 2-year group was better than that of the surgery-alone group, not only in the UFT term, but also in the S-1 (*p* = 0.0224). Longer RFS was evident in the S-1 term, especially for the 2-year group (*p* = 0.0110), and a multivariate analysis showed that both the stage of the cancer and 2 years of postoperative adjuvant chemotherapy were independent factors predictive of prolonged survival. Not only in the retrospective study but also in the randomized trial of S-1 for gastric cancer (ACTS-GC), the duration of adjuvant chemotherapy was proportional to the overall survival. Among the 517 patients in the safety population who received S-1, the persistence rate of S-1 treatment for 12 months was just 65.8 %. Besides, the dose was decreased in half of the patients who received treatment for 12 months [5]. The reasons for treatment withdrawal were patient refusal or investigator decisions due to adverse events or complications, metastasis, relapse, or presence of another cancer [5, 13]. For another examples, the recurrence of GIST is common in the initial years following discontinuation of adjuvant imatinib treatment. So more than 12 months could be reasonable for adjuvant treatment of patients with a highly estimated risk of GIST recurrence after surgery [14]. Joensuu and colleagues [15] investigated and concluded that 3 years of adjuvant imatinib administration improved RFS and OS in GIST patients with a high risk of recurrence compared to 1 year of imatinib. When it comes to women with estrogen receptor-positive breast cancer, continuing tamoxifen for 10 years rather than stopping at 5 years produces a further reduction in recurrence and mortality, particularly after 10 years [16].

However, this opinion is likely to evoke strong opposition from other studies. Colleoni M. et al. reported that 3 cycles of cyclophosphamide/methotrexate/fluorouracil (CMF) would be sufficient for women aged under 40 with hormone receptor positive, potentially endocrine responsive node-positive disease if CMF were followed by effective endocrine therapy. For women of any age with tumors that do not express any steroid hormone receptors (ER-absent) (a relatively small subgroup of patients), the issue of adjuvant chemotherapy duration requires further study, but this results do not suggest that adjuvant CMF can safely be reduced to 3 cycles in these women [17]. With regard to colorectal cancer, Des Guetz G. and his colleagues performed a meta-analysis of all RCTs comparing two durations of 5-FU-based adjuvant treatment, 6 months versus 9 to 12 months. Shorter duration of chemotherapy (3–6 months) compared with longer duration (9–12 months) was not associated to poorer RFS (RR = 0.96, 95 % CI 0.90–1.02) and OS (RR = 0.96, 95 % CI 0.91–1.02). This meta-analysis confirmed that adjuvant chemotherapy of CRC should not last for more than 6 months [18].

Besides heterogeneous regimens and inadequate medical records, this study has so many difficulties in being comprehended because the data derived from changes in chemotherapy schedule or fluoropyrimidine dosage were not directly connected to compliance in our patients. The reasons why patients were treated with no chemotherapy or with 6–12 to 24 months of chemotherapy and why patients were treated with single agent or combination were presumed to be are because of physician’s choice or intolerance to chemotherapy in patients with adverse reactions. Toxicity profiles were not recorded schematically in most patients. However, unbearable adverse events like nausea, vomiting, and peripheral neuropathy were more common in platinum-combined regimens, which were not administered for more than 6 months traditionally. No patient received 1 or 2 years of platinum-based regimen was found in this study. On the other hand, most of single oral FU-based chemotherapy regimens were administered for more than 6 months, as pointed out in the introduction, long-term oral administration of fluoropyrimidines such as doxifluridine or tegafur was commonly used as adjuvant chemotherapy for gastric cancer because the optimal period or optimal total doses of fluoropyrimidines have been rarely studied.

In the results, we found that the 3-year RFS rate is lower in the adjuvant chemotherapy group than in the surgery-only group. Perhaps, it seems like main reason that relatively more patients with high nodal stage were included in the adjuvant chemotherapy group than in the surgery only group as shown in Table 1.

There are still unresolved issues. Firstly, adjuvant chemotherapy does not prolong OS [Fig. 2a] and RFS [Fig. 2b] over surgery alone. Secondly, OS and RFS is significantly lower in the <6, 6-12 months of adjuvant chemotherapy group than in surgery only group [Fig. 3a-b]. The most likely explanation of this phenomenon is that stage III patients with poor prognosis are located dominantly in the left lower quadrant area of Table 3. Thirdly, 12-24 months of adjuvant chemotherapy significantly prolong OS and RFS at multivariate analysis, not at univariate analysis. Unfortunately, we cannot give explicit answers to these complex statistical issues except that retrospective data analysis are susceptible to bias in data selection and it may show associations among variables, but rarely establishes causal relationships [19].

## Conclusions

By this retrospective analysis, the authors did not verify that the prolonged administration of oral fluoropyrimidine derivatives as postoperative adjuvant chemotherapy could lead to a favorable outcome for stage II and III GC patients. Therefore, we propose further prospective randomized studies to determine the appropriate fluoropyrimidine-based regimens and optimal duration of adjuvant chemotherapy for higher OS and RFS in GC patients.

## Abbreviations

5-FU: 5-fluorouracil
CRC: colorectal cancer
GC: gastric cancer
RCT: randomized controlled trial
RR: relative risk
SD: standard deviation
